# Antimicrobial activity of chitosan /corn starch film incorporated with starch nanocrystals /nettle essential oil nanoemulsion for *Eleutheronema tetradactylum* fillet preservation

**DOI:** 10.1016/j.fochx.2024.102085

**Published:** 2024-12-10

**Authors:** Hamed Ahari, Fatemeh Kalateh-Seifari, Shima Yousefi

**Affiliations:** Department of Agriculture and Food Science, Science and Research Branch, Islamic Azad University, Tehran 476714171, Iran

**Keywords:** Biofilm, Antibacterial, Nanocrystals, Nanoemulsion, Fish fillet

## Abstract

This study aimed to estimate the effects of chitosan/ corn starch (CH/ CS equal 62:38) film in combination with nettle essential oil nanoemulsions (0.41 wt% NEONEs) and starch nanocrystals (6 wt% SNCs) on the microbial and qualitative characteristics of the *Eleutheronema tetradactylum*fillets during refrigeration storage (4 ± 1 °C). The fillets were covered by biopolymeric films (CH/CS, CH/CS/SNCs, CH/CS/ NEONEs, CH/CS/SNCs/NEONEs). The qualitative analysis of refrigerated fillets was performed on days 1, 7, and 10. The incorporation of NEONEs and SNCs into CH/CS made an active film with antimicrobial effects. The decrease in pH (5.89 %), PV (44.72 %), FFA (10.41 %), TVB-N (35.01 %), TBA (27.07 %) and increase in moisture (5.38 %) were observed in the covered fillets by CH/CS/SNCs/NEONEs film in compared to uncovered fillets at 4 °C on day 10. The results revealed that incorporating SNCs (6 %) and NEONEs (0.41 %) into CH/CS could increase the storage time of the refrigerated fish fillets.

## Highlights

• Chitosan: corn starch (CH/ CS) film containing nettle essential oil nanoemulsions (NEONEs) and starch nanocrystals (SNCs) were developed.
• Physicochemical properties of CH/ CS films were affected by both SNCs and NEONEs.
• CH/CS film incorporated with SNCs and NEONEs exhibited strong antibacterial activity.
• The CH/CS/SNCs/NEONEs nanocomposite film could be used as active packaging material.
• The nanocomposite films delayed the microbial growth of fish fillet.

## 1 Introduction

Biodegradable films and coatings have been suitable substitutes for synthetic films in the packaging industry due to their inherent properties and no dependency on non-renewable resources. Their characteristics have garnered interest from researchers in the packaging industry. Biodegradable films and coatings could be used as a barrier against humidity, water vapor, and other gases while they are exchanging needed gases for food respiration. Also, they are suitable for adding a wide range of additives like antifungals, antimicrobials, and antioxidants. Despite the high abundance of starch, it is not considered a suitable source for packaging films and coatings. Due to its low thermal decomposition point, high tendency to retrogradation, lack of sufficient strength, relatively low resistance, especially in humid environments, and high fragility, its use in the food industry is limited (Fan et al., 2023; Yin et al., 2023). Chitosan (CH) is a linear polysaccharide obtained through the deacetylation of chitin. Chitin can be found in the cell walls of certain algae and is a crucial component of the exoskeletons of arthropods like insects, crabs, shrimps, and lobsters. Chitosan is an attractive material for food packaging due to its biodegradability, antimicrobial properties, and ability to be tailored for improved mechanical and barrier performance. It could be used in biofilm preparation to increase the shelf life of foods, especially marine products (Rathod et al., 2023). Starch/chitosan composite biofilms offer a sustainable, cost-effective, and functional alternative to petroleum-based packaging with improved mechanical, barrier, and antimicrobial properties. Some characteristics of starch/chitosan composite biofilms (high hydrophilicity and water solubility (WS), low mechanical strength, high brittleness, and UV light sensitivity) have encouraged researchers to explore incorporating materials like nanoparticles into starch-chitosan composite films to improve their properties for food packaging applications (Wu et al., 2023).

Nowadays, biopolymer-based nanocomposites are known as a promising green alternative to conventional plastics for improving food preservation. They have unique functional characteristics needed for specific food types, including fish. The packaging characteristics required to preserve fish qualities include providing a tight moisture and gas barrier, preventing dehydration and oxidation, and inhibiting microbial growth and enzymatic spoilage. Nanocomposite biofilms and coatings could be used as a fortunate green solution to satisfy these needs and increase the shelf life of seafood products (Qiu et al., 2022). Starch nanocrystals (SNCs) are starch-based nanoparticles that have attracted considerable attention as a nanofiller for producing biopolymeric nanocomposites in food packaging industries. These crystalline nanoparticles are made by acidic or enzymatic hydrolysis and ultrasound treatment of different starch sources. Their high aspect ratio and ability to modify starch properties make them a promising nanomaterial. Due to the essential oils' antibacterial and antimicrobial properties, their incorporation in biodegradable films and coatings has been known as a potential source of novel packaging in food industries (Batool et al., 2023; Ferreira et al., 2021; Khalid, 2023). Nettle essential oil extracted from the stinging nettle plant (*Urtica dioica*) contains compounds that give it the potential to inhibit the growth of various bacteria, fungi, and viruses. Due to their unique inherent properties (high solubility, stability, and multi-targeted antimicrobial and antibacterial mechanisms), using nanoemulsion essential oils could significantly improve the biofilm's antimicrobial and antibacterial properties compared to the conventional use of essential oils (Kalateh-Seifari et al., 2021). Substantial effects of incorporating nanomaterials on the antimicrobial and functional effects of the starch–chitosan films have been reported in many researches. The starch/zein composite film containing chitosan nanoparticles infused with curcumin has been shown to delay physicochemical alterations in *Schizothorax prenati* fillets, extending their shelf life by up to 15 days (Xin et al., 2020). Also, it has been reported that incorporating 2 wt% *Thymus kotschyanus* essential oil and 1 wt% pomegranate peel extract in chitosan-starch composite film prolonged the beef's shelf-life during 21 days of storage at 4 °C (Mehdizadeh et al., 2020). Cinnamon and Ho Wood essential oils in chitosan nanocapsules loaded into starch film have been proposed for the enhancement of perishable fruits' shelf-life, without any fungi contamination (Ferreira et al., 2021).

Despite various research on the consideration of essential oils or nanocrystals on the biofilms' quality as food packaging materials, no report is available on the simultaneous evaluation of incorporation of SNCs and NEONEs into CH/CS films for enhancing the shelf life and safety of fish products. This study aimed to evaluate the effect of SNCs and nettle essential oil nanoemulsions (NEONEs) incorporation on the physical characteristics of chitosan /corn starch (CH/CS) films. We also evaluated the effect of CH/CS/ SNCs/ NEONEs on the qualitative and microbial characteristics of *the Eleutheronema tetradactylum* fillet.

## 2 Materials and methods

### 2.1 Materials

Fresh aerial parts of stinging nettle (*Urtica dioica L.*) were collected from the Chaboksar forest in Guilan, Iran. Sunflower oil was prepared from Tabiat Sabz Co., Iran. All chemicals and reagents, including acetic acid, chitosan (300 kDa, 80–90 % deacetylation), corn starch, Tween 80, sulfuric acid, ammonium thiocyanate, potassium sorbate, glycerol, and sodium sulfate were of analytical grade supplied by Merck (Darmstadt, Germany).

### 2.2 Preparation of starch nanocrystals, nanoemulsions and nettle essential oil

Starch nanocrystals powder (mean particle size equal to 102 nm) was obtained by hydrolyzing the corn starch granules followed by centrifuge, ultrasonic homogenization, and then spray drying with a lab-scale spray drier (Buchi B290, Switzerland) according to a method described in our previous study. Briefly, sulfuric acid solution (98 %) was employed for the starch granules hydrolysis under agitation (100 rpm) at 40 °C for 5 days. The resulting suspension was centrifuged (1000 rpm at 4 °C for 30 min). The supernatant was collected and washed several times with water to remove the acid residuals. The resulting pellets diluted in water and were homogenized at 24 kHz for 15 min, with an ultrasonication homogenizer (Model 3000, Biologic Inc., Manassas, VA, USA) to obtain well-distributed SNCs in water. Finally, the sonicated suspension was spray-dried using a lab-scale spray drier (Buchi B290, Flanil, Switzerland) to achieve SNCs powder (Kalateh-Seifari et al., 2021).

**Fig. 1:**
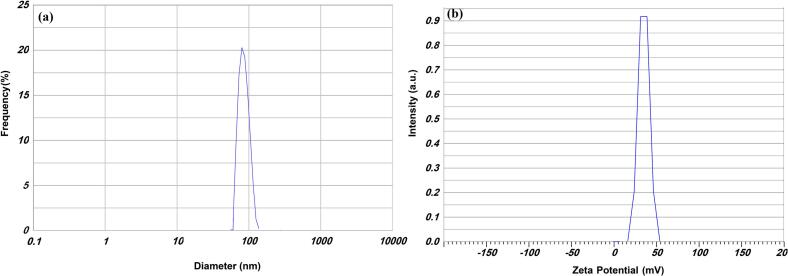
a) Average particle size diameter, b) Zeta potential of prepared nanoemulsion.

Nettle essential oil (NEO) preparation was performed by freeze-drying (Model FD-4, Pishtaz Co., Tehran, Iran) of the partially sun-dried nettle leaves. The dried leaves (500 g) were added to distilled water (2.0 L) in a flask connected to a Clevenger apparatus for 5 h at the boiling point. The obtained NEO was desiccated over anhydrous sodium sulfate and kept at 4 °C until use. The phenolic compounds in the nettle essential oil were identified using gas chromatography–mass spectrometry (GC–MS; GC 7890 N, AGILENT, and MS 5975C). A stable oil-in-water NEs was created with a ratio of aqueous to oil phase of 90 to 10 wt% (mean particle size: 90.6 nm; zeta potential: 31.5 mV) using a high-speed ultra-homogenizer (Ultraturrax T25, Germany) at 12,000 rpm for 3 min at ambient temperature (Fig. 1). The oil phase included 7.5 % sunflower oil and 2.5 % NEO, while the aqueous phase was dispersed in an acetate buffer solution (10 mM; pH 4.0) with Tween 80 serving as the surfactant. The results of our previous work were used for the preparation of biofunctional films with improved physicochemical properties (Kalateh-Seifari et al., 2021).

### 2.3 Antioxidant capacity of nettle essential oil

DPPH free-radical scavenging assay was employed for the antioxidant activity evaluation of NEO (Mahmudzadeh et al., 2019). Briefly, NEO solution with different concentrations (7.5, 15, 100, 250, and 500 μg/ml) was prepared with a definite amount of NEO to 90 μM methanolic solution of DPPH followed by incorporation of a definite amount of methanol. After 60 min, the absorbance of each solution was recorded at 517 nm using a UV spectrophotometer and was compared with the blank sample. DPPH inhibition activity of the different solutions of NEO was determined using Eq. (1).

(1) $$\text{Inhibition activity}=\frac{\left(\mathrm{Absorbance of blank}-\mathrm{Absorbance of sample}\right)}{\mathrm{Absorbance of blank}}\times 100$$

### 2.4 Antibacterial activity of nettle essential oil

The minimum bactericidal concentration (MBC) and minimum inhibitory concentration (MIC) of NEO against *Staphylococcus aureus, Salmonella enteritidis, Escherichia coli, Bacillus cereus, Pseudomonas,* and *Bacillus subtilis* were determined using microdilution method. All the bacteria were cultivated in BHI (9 ml) broth and incubated (37 °C / 24). The desired concentration of each bacterial suspension (1.5 × 106 cfu/ml) was prepared based on the 0.5 McFarland standard turbidity while in the log phase and was serially diluted (1:10). The stock solution (40 mg/ml) was created by dissolving NEO in 10 % dimethyl sulfoxide, followed by serial two-fold dilutions (0.31–40 mg/ml) using distilled water (DW). In 96-well microplates, 20 μl of each bacterial suspension (1.5 × 106 cfu/ml) and 160 μl of BHI broth were added. Then, 20 μl of the stock solution was added to the first wells, and subsequently, 20 μl of the serial two-fold dilutions were added to the following wells. The negative control was created using 180 μl of BHI broth and 20 μl of NEO, while the positive control was made with 180 μl of BHI broth and 20 μl of the inoculum. Ultimately, the NEO concentrations ranged from 0.031 to 4 mg/ml, and the concentration of the bacterial suspensions was approximately 1.5 × 105 cfu/ml. The MIC was defined as the lowest concentration of NEO that showed no *v*isible bacterial growth. The MBC values of NEO were determined by inoculating 10 μl from the non-turbid wells onto BHI agar and assessing the minimum concentrations with no *v*isible bacterial growth (Mirahmadi et al., 2020).

### 2.5 Nanocomposite film preparation

**Fig. 2:**
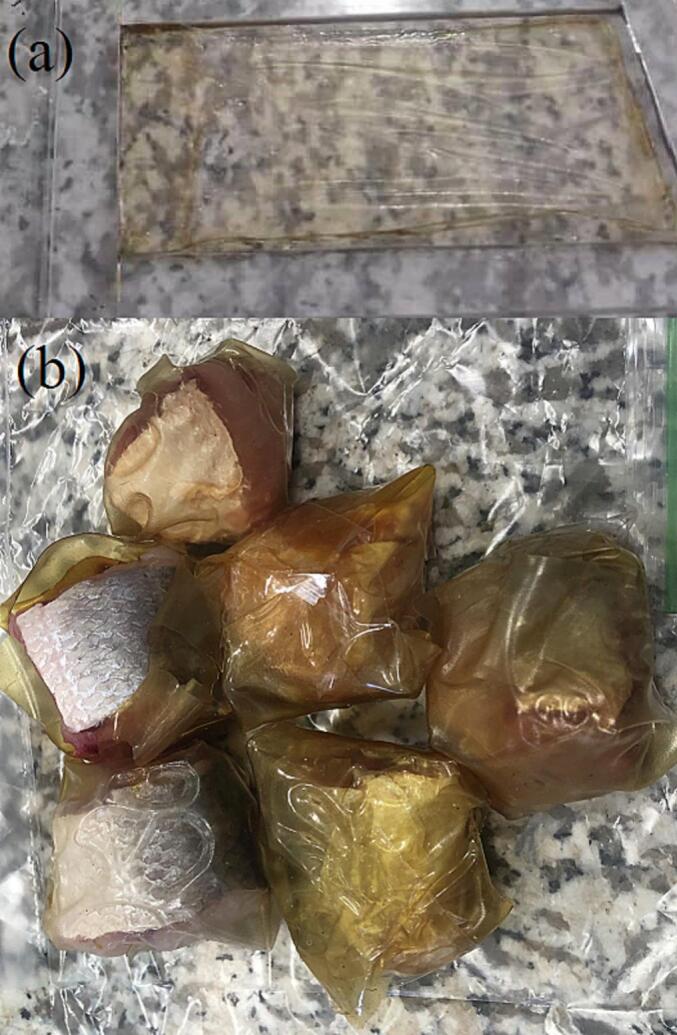
a) CH/CS/SNCs/NEONEs nanocomposite film, b) the wrapped *Eleutheronema tetradactylum* fillet in the nanocomposite film.

Biopolymer composite films were fabricated using the solvent casting method, incorporating chitosan and corn starch in a ratio of 62:38 (CH: CS), along with nettle essential oil nanoemulsions (NEONEs at concentrations of 0 % and 0.41 %) and starch nanocrystals (SNCs at concentrations of 0 % and 6 %) (Table 1). In summary, a chitosan solution was prepared by dissolving chitosan biopolymer (2 % *w*/*v*) in a 1 % *v*/v acetic acid solution on a hotplate with a magnetic stirrer, maintaining a speed of 300 rpm and a temperature of 70 °C for 24 h. The solution was created by dissolving corn starch (3.5 % w/v) in double-DW on a hotplate with a magnetic stirrer at 300 rpm and 90 °C for 30 min. The composite films made from corn starch and chitosan (with a ratio of CH: CS equal to 62:38) were produced by combining a specific amount of chitosan with a corn starch solution. Glycerol was added as a plasticizer at 25 % (*w*/w) of the total dry matter. A specified quantity of NEONEs and SNCs was incorporated into the final solution and homogenized at 7000 rpm for 3 min. The resulting solution was cast into Teflon plates and set for 72 h at ambient temperature before being peeled off. The nanocomposite films were then exposed to a relative humidity (RH) of 50 % for 72 h before application (Fig. 2a).

**Table 1:** Chitosan: corn starch (CH:CS equal 62:38) films.

Treatment	SNCs (%)	NEONEs (%)
Control	0	0
T1	6	0
T2	0	0.41
T3	6	0.41

### 2.6 Composite films characterization

#### 2.6.1 Water solubility

The WS of the films was assessed according to (Mehdizadeh et al., 2020) with some modifications. Briefly, the films were cut into squares measuring 2 cm on each side and dried overnight in an oven at 60 °C, after which their initial dry weight (W1) was recorded. The dried films were then placed into boiling tubes for 1 h and subsequently dried again for 24 h in an oven at 60 °C. After this drying period, the final weight of the samples was measured (W2), and the WS was calculated using Eq. (2).

(2) $$\mathrm{Water solubility}\;\left(\%\right)=\frac{W_{1}-W_{2}}{W_{1}}\times 100$$

#### 2.6.2 Water absorption capacity (WAC)

The WAC of the composite films was assessed according to (Sadegh-Hassani & Nafchi, 2014). The films were dried in phosphorus pentoxide for 7 days. Afterward, samples were cut into squares measuring 2 cm on each side and immersed in DW (100 ml) for 30 min at 23 °C. At the end of this period, the surfaces dried swollen films with a cotton cloth were weighed and the absorbed water was recorded as WAC.

#### 2.6.3 **Oxygen permeability** (OP)

The OP of the films was measured using a permeance testing apparatus (GDP-C, Germany) (Kalateh-Seifari et al., 2021). Composite films measuring 2 cm × 2 cm were positioned between the chambers of the apparatus. The upper chamber was filled with food-grade oxygen at atmospheric pressure, and gas permeation was assessed by measuring the pressure increase in the lower chamber. The experiments were conducted with a gas flow rate of 100 cm^3^/min, a RH of 0 %, and a film area of 0.650 cm^2^.

#### 2.6.4 Mechanical analysis of the composite films

The tensile strength (T) and elongation at break (EB) of the composite films were assessed through mechanical analysis using an Instron tensile tester (5542, USA) set at a crosshead speed of 10 mm/min and an initial grip separation of 50 mm. The samples were cut into pieces measuring 10 cm × 0.5 cm and stored in a desiccator at 50 % RH at ambient temperature for 24 h to achieve moisture equilibrium.

#### 2.6.5 Microstructural and morphological analysis of the nanocomposite films

The chemical composition and functional groups of the nanocomposite films were evaluated using Fourier transform infrared (FTIR, Perkin Elmer, Inc., USA) spectrum. The crystalline structure of the nanocomposite films was identified with an X-ray diffraction (XRD, Shimadzu Co., Japan) pattern. The morphological characteristics of the prepared nanocomposite films were evaluated using a transmission electron microscope (TEM, Philips CM120, Netherlands) and scanning electron microscopy (SEM, TESCAN MIRA3, Czech Republic).

### 2.7 Performance of selected composite film on the fish fillet

The fresh *Eleutheronema tetradactylum* fish was obtained from the seawater of Bushehr port (South part of Iran). The fish were sent to the laboratory in a box packed with ice cubes, immediately. The fishes were washed, and 50 g of the prepared fillet was wrapped up in the selected composite film (Fig. 2b). The *Eleutheronema tetradactylum* fillet without co*v*er was considered as a control fillet sample (6 pieces of fillets for each sample). Each sample was labeled and placed into sterile petri dishes, and stored at 4 °C for 1, 7, and 10 days.

#### 2.7.1 Qualitative and microbial characteristics of fish fillets

At each sampling day, pH, moisture, peroxide, free fatty acid, TVB-N, TBA, and microbial load were evaluated using the following methods. A digital pH meter (Metrohm 827 pH Lab, Switzerland) measured the pH of each homogenized sample in distilled water at room temperature. The percentage of fillet weight loss was measured in an oven at 103 °C for moisture content determination Peroxide values were assessed by dissolving fish fillets in acetic acid-chloroform solution (3:2 *v*/v), addition of potassium iodide solution, and titrating using a sodium thiosulfate standard solution (25 g/L). In contrast, the starch solution was used as an indicator. The peroxide value was calculated with the procedure described by (Monirul et al., 2019). The free fatty acid (FFA) content was estimated in terms of oleic acid using titration of the mixture of the cold press extracted oil and ethanol with a few drops of 1 % phenolphthalein by 0.1 N NaOH according to the method described by (Mirzapour-Kouhdasht & Moosavi-Nasab, 2020). Thiobarbituric acid index (TBA) was measured based on spectrophotometry of the pink complex obtained from the reaction of malondialdehyde and thiobarbituric acid-reactive substances (TBARS) at 532 nm and described as mg MDA/kg. Total volatile basic nitrogen (TVB-N) was measured using titration of the solution containing 2 % boric acid, some drops of methyl red, and the distilled minced samples (obtained from the Kjeldahl distillation method) with 0.1 N sulfuric acid solution (Mirzapour-Kouhdasht & Moosavi-Nasab, 2020). Microbial analysis was performed using psychrophilic bacterial counts (PTC) and total viable count (TVC) measurement by standard plate count method on the PCA culture medium with the procedure described by (Han et al., 2015).

#### 2.7.2 Sensory analysis

The sensorial properties of the fish fillet samples were evaluated by five experienced panelists. The sensory parameters of fish fillets including aroma, color, texture, and overall acceptance were considered. The 5-point scale hedonic scores (5: excellent, 4: good, 3: fair, 2: poor, and 1: very poor) were used for the parameters evaluation (Furey et al., 2024). https://doi.org/10.1080/15428052.2022.2027308

### 2.8 Statistical analysis

The experiments were conducted in triplicate and mean and standard deviation values were expressed for the results. One-way analysis of variance was carried out on the test results to evaluate the significant difference between samples (*P* < 0.05). Duncan's test was conducted to compare means using SPSS 18.0.

## 3 Results and discussion

### 3.1 Antioxidant and antibacterial activity of the extracted NEO

The IC50 (half-maximal inhibitory concentration) represents the concentration of an antioxidant for scavenging 50 % of the free radicals or oxidants present in the system. It is commonly used to assess the efficacy of antioxidants, particularly in DPPH radical scavenging assays. A lower IC50 value indicates that a smaller antioxidant concentration is needed to achieve 50 % inhibition of the free radicals. According to the results, the NEO displayed a significant DPPH radical inhibition, with IC50 at 204.7 μg/ ml compared to 269.5 μg/ml for the standard ascorbic acid. The presence of phenolic compounds is responsible for the singlet oxygen and free radicals scavenging (Ebrahimi et al., 2024). The GC–MS analysis showed the phenolic compounds of nettle essential oil (2-chlorophenol, Phenol, 2,4-dimethyl phenol, 2,4,6-trichlorophenol, 2,4-dichlorophenol, 4-chloro-3-methyl phenol, 2-nitrophenol, 4-nitrophenol, Pentachlorophenol equal to 9.9, 17.7, 6.8, 8.7, 47.7, 8.1, 15.3, 17 and 23.5 ppb, respectively). The phenolic compounds of stinging nettle (*Urtica dioica L.*) and their antioxidant effect were reported in different studies (Đurović et al., 2024). The results of the MIC and MBC evaluation of the extracted NEO against the considered bacteria are shown in Table 2. The results revealed that the extracted NEO demonstrates antibacterial activity against *Staphylococcus aureus, Escherichia coli, Bacillus cereus, Salmonella enteritidis, Pseudomonas,* and *Bacillus subtilis*, with MIC and MBC values in the range of 1.16–13.33 and 10–16.66 mg/ml, respectively. However, *Salmonella Enteritidis* was more resistant, requiring nettle extract concentrations greater than the concentration needed to inhibit the other considered microorganisms' growth. Strong antimicrobial activity of nettle oil extract was reported in different studies which is consistent with the present research (Gülhan and Yangılar, 2022, Gülhan and Yangılar, 2024; Kőszegi et al., 2017).

**Table 2:** MIC and MBC of the extracted NEO against the considered bacteria.

Microorganisms	MIC (mg/ml)	MBC (mg/ml)
*Escherichia Coli*	10	16.66
*Samonella Enteritidis*	13.33	16.66
*Pseudomonas*	10	13.33
*Staphylococcus Aureus*	4.16	10
*Bacillus Cereus*	8.33	10
*Bacillus Subtilis*	10	13.33

**Fig. 3:**
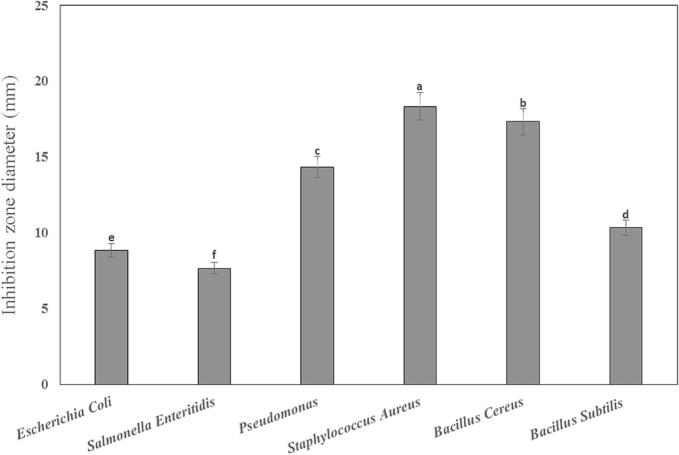
The inhibition zone diameter of NEO against considered bacteria. * Different letter means significant difference between means.

The specific inhibition zone diameters can provide insights into the potential use of the NEO in antimicrobial applications. As shown in Fig. 3, the inhibition zone diameter of NEO against considered bacteria in BHI broth varied significantly depending on the specific bacterial strain tested (*P* < 0.05). It is confirmed in the antibacterial effect evaluation of nanoemulsion and nanoencapsulation of a hydroethanolic extract of Nettle (*Urtica dioica*) in BHI media (Rahmani et al., 2024).

### 3.2 Films characterization

**Fig. 4:**
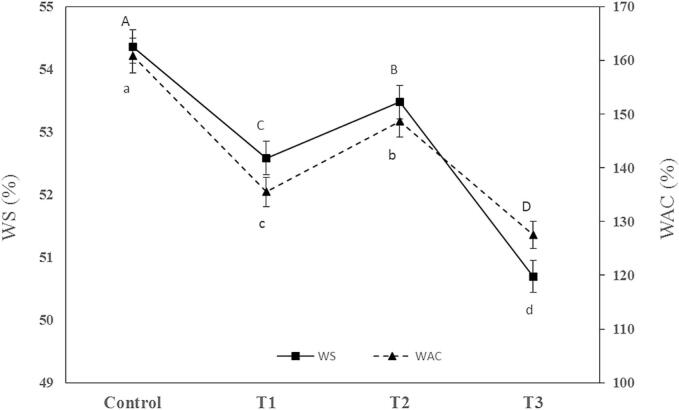
Water solubility (WS) and water absorption capacity (WAC) of treatments. * Different letter means significant difference between means.

The results of WS and water absorption capacity (WAC) measurements are shown in Fig. 4. The WS and absorption capacity of biodegradable food packaging plays a crucial role in its environmental impact, functionality, and consumer convenience. They are key properties for maintaining the integrity and freshness of food products over time. In fish packaging, the lower water solubility of films enhances their stability when in contact with moist fish products. This stability is essential to prevent the film from dissolving or degrading during storage, which could lead to contamination or spoilage of the fish (Athanasopoulou et al., 2024). The results of WS and WAC evaluation revealed the presence of 6 wt% SNCs in CH/CS composite film decreased WS and WAC by about 3.2 % and 15.75 %, respectively (P < 0.05). Also, the presence of 0.414 wt% NEONEs decreased WS and WAC by about 1.6 % and 7.58 %, respectively (P < 0.05). While the simultaneous presence of 6 wt% SNCs and 0.414 wt% NEONEs decreased WS by about 6.7 % and WAC by about 20.75 % (P < 0.05). The use of SNCs leads to a rise in the crystallinity of the films, which impacts their water solubility (Singh et al., 2023). More crystalline structures have less tend to dissolve in water. Nevertheless, the effect could vary depending on the concentration and type of used SNCs. Also, the SNCs addition could facilitate hydrogen bonding within the film. It could enhance the structural integrity and reduce water solubility (Martins et al., 2022). However, SNCs reinforced films generally show lower water absorption capacity compared to non-reinforced films. This indicates that not only SNPs could improve the films' structural integrity, but also they may reduce the overall water absorption capacity. It is useful for maintaining the quality of packaged food. The decrease in water solubility of essential oil nanoemulsions-loaded films could be attributed to the hydrophobic nature of the oils and their capability to make a tight and dense film structure. However, it leads to the formation of less water-soluble films (Rui et al., 2024).

A decrease in water solubility of the carboxymethyl chitosan/locust bean gum composite films with increasing concentrations of essential oil nanoemulsions was reported by (Yu et al., 2022). Also, the decrease in the water solubility of chitosan film was reported by adding clove essential oil (CEO) NE to the chitosan films (Rui et al., 2024). Oxygen permeability, as a critical factor in food packaging, influences the quality and shelf life of products which are significantly affected by film composition (Zabihzadeh Khajavi et al., 2020). The results have demonstrated that the NEONEs addition to the CH/CS film had no significant effect on the oxygen permeability (*P* > 0.05). While the addition of SNCs to the CH/CS film significantly reduced the oxygen permeability of CH/CS film by about 5.96 % (from 3.53 to 3.32 (meq/kg O2)) (*P* < 0.05). Also, the simultaneous addition of SNCs and NEONEs significantly reduced the OP by about 13.6 % (from 3.53 to 3.05 (meq/kg O2)) (P < 0.05). It could associated with the synergistic effect of the simultaneous addition of SNCs and NEONEs on the OP of the film. he CH/CS/SNCs/NEONEs film has shown significant oxygen barrier performance improvements, reducing oxygen transmission rates by creating tortuous paths for oxygen gas molecules, thus enhancing preservation capabilities. Studies have shown that the starch nanoparticles incorporation can significantly lower the OP of various polymer matrices, making them more effective for food preservation (Sharafi Zamir et al., 2022; Singh et al., 2022). It is reported that the essential oils addition to biopolymeric films could alter the film's crystallinity. It could affect the gas barrier properties due to changes in the film crystallinity (Zubair et al., 2022). The results revealed that the NEONEs and SNCs incorporation into the CH/CS film significantly influences their tensile properties, including elongation at break (EB) and tensile (T). The tensile enhanced with the addition of SNCs and NEONEs, while the EB decreased in the case of the SNCs addition and increased in the case of the NEONEs addition. The EB decrease in the case of the SNCs addition is attributed to the increased rigidity of the films due to the presence of nanocrystals, which restricts the ability of the material to stretch before breaking (Niranjana Prabhu & Prashantha, 2018). The simultaneous addition of SNCs and NEONEs to the CS/CH film resulted in a nanocomposite film with an EB of 53.54 %, and T of 0.20 MPa.

### 3.3 Microstructural and morphological analysis of the nanocomposite films

**Fig. 5:**
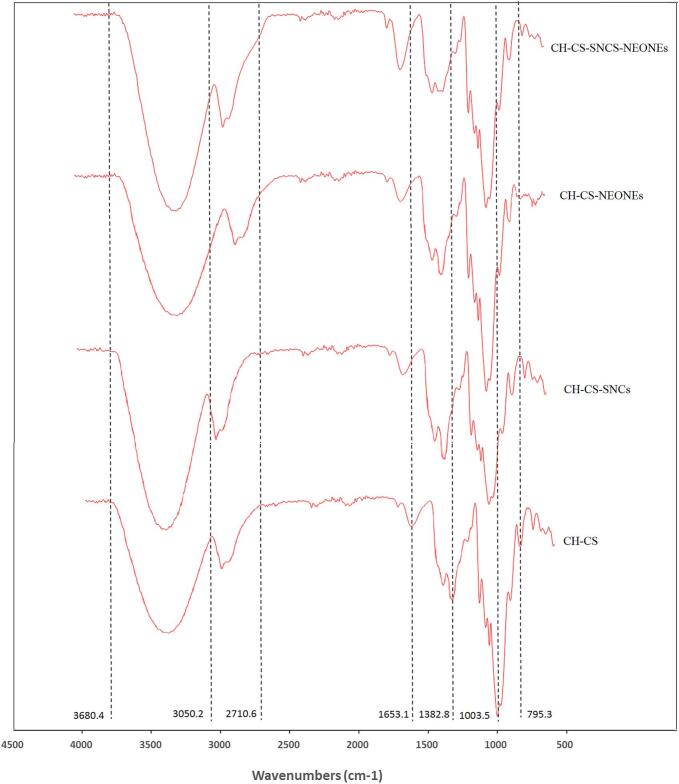
The FTIR spectra of CH/CS (Control), CH/CS/SNCs (T1), CH/CS/NEONEs (T2), CH/CS/ SNCs/NEONEs (T3).

FTIR spectra provide information about the molecular structure and composition of materials. The FTIR spectra of the four prepared films (Table 1) are shown in Fig. 5. A broad absorption peak around 3200–3600 cm^−1^ corresponds to -OH stretching, indicating inter- and intra-molecular hydrogen bonding between starch, chitosan, and other components including glycerol, SNCs, and NEONEs (Wiwattanapatapee et al., 2024). This peak shifts to lower wavenumbers due to the incorporation of SNCs and NEONEs in CH/CS composite film compared to pure starch, suggesting the formation of new hydrogen bonds upon incorporation of the essential oil (Cai et al., 2020). Absorption bands in the region of 2800–3000 cm^−1^ are associated with —CH_2_ stretching vibrations and both symmetric and asymmetric stretches of the C—H bonds, reflecting the aliphatic chains present in both starch and NEONEs, which are significant in starch molecules (Krajewska & Mietlińska, 2022). Chitosan exhibits absorption bands from 1680 to 1480 cm^−1^ due to amide-I stretching and N—H bending. In the blend films, these peaks appeared in 1653 cm^−1^ and 1480 cm^−1^, indicating interactions between the amide groups of chitosan and other functional groups. Additional peaks at approximately 1003, 1075, and 993 cm^−1^ are related to the stretching vibrations of C—O bonds, C—O— C, and C-O-H groups found in the anhydrous glucose units of starch (Smith, 2022). Specific peaks such as those around 790 cm^−1^ indicate aromatic C—H bending, confirming the presence of aromatic compounds from nettle essential oil in the starch films (Bhandari et al., 2017). The FTIR spectra of CH/CS/SNCs/NEONEs film show shifts in peak positions and variations in intensity compared to CH/CS film, suggesting effective interactions and blending of these materials.

**Fig. 6:**
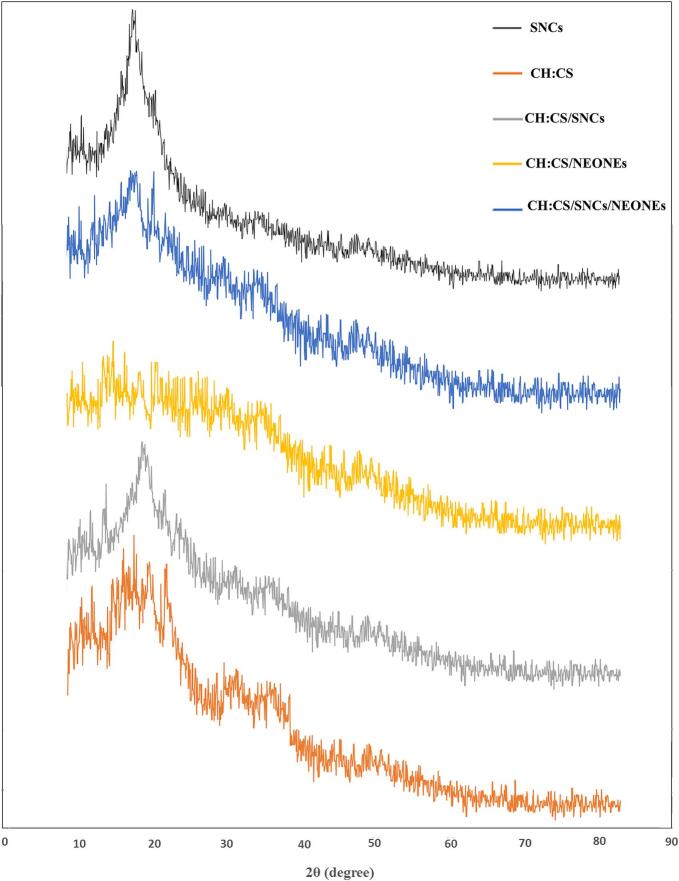
The XRD patterns of CH/CS (Control), CH/CS/SNCs (T1), CH/CS/NEONEs (T2), CH/CS/ SNCs/NEONEs (T3).

The XRD analysis of the four prepared films containing starch/chitosan/SNCs and NEONEs blend film reveals important information about their structure. The XRD pattern of CH/CS blend films containing SNCs and NEONEs is shown in Fig. 6. XRD analysis was done to examine the change in crystalline and amorphous regions of the blend by the addition of SNCs and NEONEs. SNCs exhibit a sharp diffraction peak around 2θ ≈ 18°. This peak indicates the crystalline structure of SNCs (Kalateh-Seifari et al., 2021; Li, Zhao, et al., 2022). The CH/CS composite film exhibited diffraction peaks at 2θ ≈ 16°, 18°and 21° and two broad peaks at 2θ ≈ 30° and 36° (Jha, 2021). When SNCs are added the peaks in the 2θ range of 16° to 21° were shifted to lower angles suggesting a more crystalline structure due to the nanocrystal interaction with the polymer matrix (Duan et al., 2011). Also, a decrease in peak width and an increase in the peak intensity were observed indicating an increase in crystallinity and more ordered structure (Hassan et al., 2022). When NEONEs were added to the CH/CS blend film, the XRD patterns showed a decrease in the peak intensity of the peaks in the 2θ range of 16° to 21°. The interaction between NEONEs and CH/CS can lead to a broadening of peaks, suggesting an increase in amorphosity (Mehdizadeh et al., 2020). This broadening indicates a decrease in crystallinity, which occurs due to the generation of hydrogen bonds between the amine groups of chitosan and the hydroxyl groups of NEONEs. Such interactions disrupt the orderly arrangement of the polymer chains, causing a more amorphous structure (Jha, 2021). The XRD pattern of CH/CS/SNCs /NEONEs shows a shift in diffraction peaks and a decrease in peak width in the 2θ range of 16° to 21° compared to CH/CS. Also, a reduction in peak intensity was observed compared to CH/CS/SNCs. It could be attributed to changes in chain orientation and the new intermolecular interaction formation due to the simultaneous addition of SNCs and NEONEs (Grande Tovar et al., 2020).

**Fig. 7:**
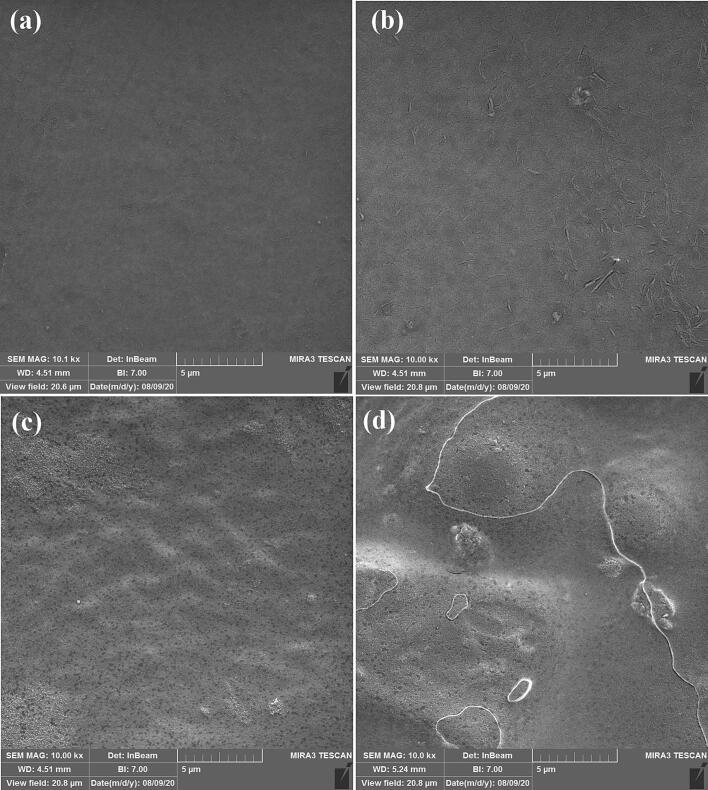
Scanning electron microscopy (SEM) Image of a) CH/CS (Control), b) CH/CS/SNCs (T1) c) CH/CS/NEONEs (T2), d) CH/CS/ SNCs/NEONEs (T3).

Fig. 7 displays the SEM images of the four films that were prepared. It revealed that the SNCs and NEONEs addition into the CH/CS film leads to a more heterogeneous surface morphology. Previous research reported that the nanocrystals disrupt the uniformity of the polymer matrix, creating a more textured surface (Adeyeye et al., 2023). Fig. 7b indicates the CH/CS/SNCs indicate the well-dispersion of SNCs into the CH/CS film. Also, the roughness presented in Fig. 7c compared to CH/CS film is attributed to the dispersed oil droplets presence. By simultaneous addition of SNCs and NEONEs to the CH/CS film, the film exhibits distinct micro-scaled features and a rougher surface Fig. 7d. These heterogeneities are characterized by increased roughness and the presence of protuberances, which suggests new regain formation within the composite film due to the formation of new intermolecular interactions (Souza et al., 2021).

**Fig. 8:**
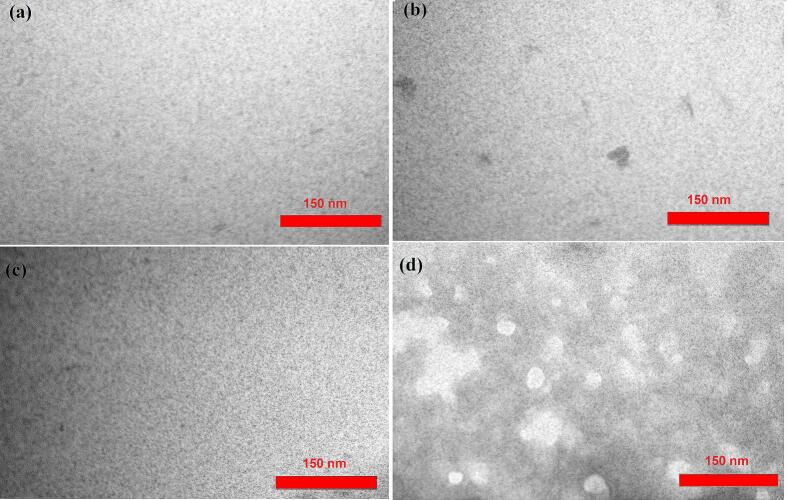
Transmission Electron Microscopy (TEM) Image of a) CH/CS (Control), b) CH/CS/SNCs (T1) c) CH/CS/NEONEs (T2), d) CH/CS/ SNCs/NEONEs (T3).

The TEM images of the four prepared films are shown in Fig. 8. The surface of the CH/CS film with the addition of SNCs has more protuberances compared to the CH/CS film containing NEONEs (Fig. 8b & c). The simultaneous addition of SNCs and NEONEs induces morphological changes. This could be attributed to new regain formation within the composite film due to the new intermolecular interactions formation (Fig. 8d). The SNCs addition into the biopolymer films usually increases crystallinity. This increase in crystallinity is visible in the TEM images. The crystalline areas seem more pronounced, suggesting a more arranged structure within the film matrix (Martins et al., 2022).

### 3.4 Qualitative and microbial characteristics of fish fillets

According to the results CH/CS/SNCs/NEONEs film was selected for the performance evaluation on the *Eleutheronema tetradactylum* fillet. The selection of CH/CS/SNCs/NEONEs nanocomposite film was due to the lower water solubility, water absorption capacity, and better oxygen barrier and mechanical properties. The results of qualitative characterizations including pH, moisture, peroxide, free fatty acid, TVB-N, and TBA of *Eleutheronema tetradactylum* fillets (covered by CH/CS/SNCs/NEONEs nanocomposite film and without cover) were shown in Table 3. The results revealed that the pH of the fish fillets (covered by CH/CS/SNCs/NEONEs nanocomposite film and without cover) changed significantly during storage over time (*P* < 0.05). However, the pH change of the uncovered fillet was higher than the covered fillet (P < 0.05). No significant difference was detected in the pH value of the covered fillet on days 7 and 10 (*P* > 0.05). The pH increase could be related to the accumulation of alkaline compounds, like amines and ammonia, which are generated by microbial action as the fish begins to spoil (Kim et al., 2023). This change demonstrates a critical indicator of fish freshness and quality, with implications for shelf life and safety (Tavassoli et al., 2024). The results showed that employing the nanocomposite film reduced the pH increases and preserved the quality of fish fillet. Fresh fish fillets had high moisture content, around 72 % at the beginning of storage. During storage, the moisture content of fillets decreased due to desiccation and drip loss (*P* < 0.05). The results revealed the moisture reduction in covered fillets was about 5.15 % lower than in uncovered ones. The peroxide value of fish fillets is a crucial indicator of lipid oxidation and quality deterioration during storage. Fresh fillets start with low peroxide values, indicating minimal oxidation. As storage progresses, the peroxide value generally increases significantly (*P* < 0.05). The rate of increase in peroxide value of the uncovered fillet was higher than the covered fillet (P < 0.05). Previous studies report various ranges of peroxide values depending on fish species and storage conditions. For fish fillets, a peroxide value below 10 meq/kg is considered acceptable, indicating good quality. Values exceeding this threshold suggest deterioration and rancidity.

**Table 3:** Qualitative characterizations of *Eleutheronema tetradactylum* fillets (covered by CH/CS/SNCs/NEONEs nanocomposite film and without cover) during storage at 4 °C.

Properties		Fillet with cover (Yes) Fillet without cover (NO)	Storage time		
			Day 1	Day 7	Day 10
pH		No	6.03 ± 0.01^Ca^	6.34 ± 0.02^Ba^	6.62 ± 0.01^Aa^
		Yes	6.07 ± 0.01^Ca^	6.14 ± 0.07^Ab^	6.23 ± 0.04^Ab^
Moisture (%)		No	72.61 ± 0.70^Aa^	68.73 ± 0. 40^Bb^	65.34 ± 0.30^Cb^
		Yes	72.62 ± 0.60^Aa^	71.65 ± 0.50^Ba^	68.86 ± 0.60^Ca^
Peroxide value (meq/kg)		No	0.300 ± 0.002^Ca^	1.181 ± 0.057^Ba^	1.583 ± 0.076^Aa^
		Yes	0.300 ± 0.002^Ba^	0.831 ± 0.057^Ab^	0.875 ± 0.025^Ab^
Free fatty acid		No	2.821 ± 0.001^Ca^	4.324 ± 0.162^Ba^	9.024 ± 0.282^Aa^
		Yes	2.821 ± 0.001^Ca^	3.760 ± 0.057^Ab^	8.084 ± 0.325^Ab^
TVB-N (mg N/100 g)		No	5.22 ± 0.16^Ca^	25.57 ± 0.85^Ba^	37.33 ± 1.16^Aa^
		Yes	5.22 ± 0.16^Ca^	20.53 ± 0.64^Bb^	24.26 ± 0.85^Ab^
TBA (mg MDA/kg)		No	0.494 ± 0.012^Ca^	0.770 ± 0.027^Ba^	1.178 ± 0.018^Aa^
		Yes	0.494 ± 0.012^Ca^	0.686 ± 0.010^Ba^	0.859 ± 0.014^Ab^
Color parameters	L*	No	68.38 ± 0.34^Aa^	65.08 ± 0.06^Bb^	62.52 ± 0.03^Cb^
	L*	Yes	68.12 ± 0.14^Aa^	66.24 ± 0.05^Ba^	63.11 ± 0.09^Ca^
	a*	No	7.26 ± 0.06^Aa^	7.06 ± 0.13^Aa^	7.09 ± 0.03^Aa^
	a*	Yes	7.27 ± 0.01^Aa^	7.20 ± 0.07^Aa^	7.16 ± 0.11^Aa^
	b*	No	10.46 ± 0.04^Ca^	11.48 ± 0.01^Ba^	13.24 ± 0.27^Aa^
	b*	Yes	10.45 ± 0.07^Ca^	11.20 ± 0.12^Bb^	12.82 ± 0.33^Ab^

*Different small latter means significant difference of the properties between covered and uncovered fillet (*P* < 0.05).

**Different capital latter means significant difference of the properties in different days (P < 0.05).

The free fatty acid content of fish fillets is an important indicator of lipid hydrolysis and quality deterioration during storage. Fresh fillets have low free fatty acid levels, indicating minimal lipid hydrolysis. As storage progresses, these levels increase significantly due to the activity of lipases, which break down triglycerides into free fatty acids (Xie et al., 2023). The results revealed that the rate of increase in the free fatty acid content of the uncovered fillets was higher than the covered fillets (*P* < 0.05). It could be associated with the lower breakdown of triglycerides into free fatty acids due to covering fish fillets with the nanocomposite film (Yi & Xie, 2021). The TVB-N content in fish fillets is another parameter for assessing fish quality and freshness during storage. TVB-N is primarily composed of amines and ammonia, which are produced as a result of microbial activity and protein degradation. Fresh fish fillets had low TVB-N values (5.22 mg N/100 g) on day 1. There was a significant increase in the TVB-N content of fillets during storage (*P* < 0.05). This increase is indicative of spoilage and the breakdown of proteins into nitrogenous compounds due to microbial and enzymatic activity. The TVB-N levels in uncovered fillets rose to about 37.33 mg N/100 g following 10 days of storage at 4 °C. The TVB-N content increased by about 24.26 mg N/100 g for the covered fillets with nanocomposite film. European Commission has set specific TVB-N limits for various fish species ranging from 20 to 35 mg N/100 g. Fish exhibiting a TVB-N level of >35 mg N/100 g are generally considered spoiled and unfit for consumption (Dergal et al., 2013). The change in TBARS levels in fish fillets during storage indices the lipid oxidation, which affects the quality and freshness of the fish. Fresh fish fillets had low TBARS values (0.494 mg MDA/kg) on day 1. TBARS levels significantly increase during storage, particularly in the uncovered fillet during storage time (*P* < 0.05). The TBARS values of uncovered fillets increased about 1.37 times more than the TBARS values of covered fillets over a storage period of 10 days, indicating more progressive lipid oxidation in uncovered fillets. A TBARS value of 5 mg MDA/kg is often considered the upper limit for acceptable quality in fish. Values exceeding this threshold suggest significant lipid oxidation and deterioration in quality (Suárez-Medina et al., 2024). The significant change in the color parameters (L*, a*, b*) of fish fillets during storage time, reflects the quality deterioration. The L* value, which represents lightness decreased slightly during storage in covered and uncovered fish fillets, indicating the fish fillets become darker. However, the decrease in the covered fillets was lower than in the uncovered fillets (*P* < 0.05). The redness value (a*) of covered and uncovered fillets had no significant change during storage indicating no significant loss of redness and a shift towards greener hues (*P* > 0.05). The yellowness value (b*) of covered and uncovered fillets had a significant increase during storage (P < 0.05). The yellowness value increment was higher in uncovered fillets.

The results of microbial load evaluation including total mesophilic count and total psychrophilic count are shown in Table 4. During storage at 4 °C, mesophilic bacterial count increased significantly for covered and uncovered fish fillets (P < 0.05). The increase in the overall mesophilic count of uncovered fish fillets was higher than covered fillets during days 7 to 10. The mesophilic bacterial spoilage threshold is generally considered to be around 7.0 log CFU/g. When mesophilic bacterial counts reach this level, the fish is typically deemed spoiled and unfit for consumption (Fernández-Saiz et al., 2013). Total psychrophilic count evaluation of fish fillets revealed the total psychrophilic count increased significantly for covered and uncovered fish fillets, during storage time (P < 0.05). The total psychrophilic count increase of uncovered fish fillets was higher than the covered fillets on days 7–10. Psychrophilic bacteria, which thrive at low temperatures, can significantly influence the shelf life of refrigerated fish. A microbiological criterion for quality and safety in refrigerated fish fillets is often established at 6–7 log CFU/g. Counts above this threshold indicate spoilage and reduced acceptability (Erkan et al., 2006). Increased oxygen levels can promote the growth of aerobic bacteria and molds, further compromising food safety and quality. For instance, studies have shown that films with high oxygen transmission rates can enhance microbial activity on packaged foods, leading to quicker spoilage (Gürdal & Çetinkaya, 2024).

**Table 4:** Microbial load of *Eleutheronema tetradactylum* fillets at 4 °C (covered by CH/CS/SNCs/NEONEs nanocomposite film and without cover) during storage at 4 °C.

Total count log (CFU/g)	Fillet with cover (Yes) Fillet without cover (NO)	Storage time		
		Day 1	Day 7	Day 10
Mesophilic	No	2.10 ± 0.28^Ca^	4.75 ± 0.63^Ba^	12.95 ± 0.21^Aa^
	Yes	2.15 ± 0.33^Ca^	4.50 ± 0.70^Ba^	5.75 ± 0.77^Ab^
Psychrophilic	No	2.75 ± 0.35^Ca^	4.10 ± 0.28^Ba^	4.90 ± 0.56^Aa^
	Yes	2.70 ± 0.42^Ba^	2.95 ± 0.21^Bb^	3.50 ± 0.13^Ab^

*Different small latter means significant difference of the properties between covered and uncovered fillet (P < 0.05).

**Different capital latter means significant difference of the properties in different days (P < 0.05).

As shown in Table 5, the organoleptic properties of the fish fillets, including color, texture, and flavor, undergo significant changes during storage at 4 °C (*P* < 0.05). This study has highlighted how nanocomposite film influenced these properties, enhancing the quality and shelf life of the fish. Flavor is a critical organoleptic property that could be varied during storage. The results revealed that the nanocomposite film helped to maintain flavor quality which could be related to limiting the degradation of volatile compounds that contribute to taste. Coverage of fish fillets with nanocomposite film exhibits less flavor loss compared to those without coverage. The nanocomposite film slows down the oxygen permeability and moisture loss, thus decreasing the metabolic processes progress that leads to flavor deterioration (Li, Tu, et al., 2022). An increase in peroxide value is associated with off-flavors and rancidity, adversely affecting the sensory qualities and shelf life of fish fillets. The results of the peroxide value evaluation of the samples revealed that the rate of increase in the peroxide value of the uncovered fillet was significantly higher than the covered fillet.

**Table 5:** The organoleptic properties of the fish fillets during storage at 4 °C.

Organoleptic properties	Fillet with cover (Yes) Fillet without cover (NO)	Storage time		
		Day 1	Day 7	Day 10
Flavor	No	4.66 ± 0.57^Aa^	3.00 ± 0.00^Bb^	2.33 ± 0.57^Cb^
	Yes	4.66 ± 0.57^Aa^	4.00 ± 0.00^Ba^	3.66 ± 0.33^Ca^
Color	No	5.00 ± 0.00^Aa^	4.00 ± 0.00^Ba^	2.33 ± 0.57^Cb^
	Yes	5.00 ± 0.00^Aa^	4.00 ± 0.00^Ba^	3.33 ± 0.57^Ca^
Texture	No	5.00 ± 0.00^Aa^	3.00 ± 0.00^Bb^	2.66 ± 0.33^Cc^
	Yes	5.00 ± 0.00^Aa^	3.66 ± 0.33^Ba^	3.33 ± 0.57^Ca^
Overall acceptance	No	4.66 ± 0.57^Aa^	3.33 ± 0.57^Bb^	2.33 ± 0.57^Cb^
	Yes	4.66 ± 0.57^Aa^	4.33 ± 0.57^Ba^	3.66 ± 0.33^Ca^

*Different small latter means significant difference of the properties between covered and uncovered fillet (P < 0.05).

**Different capital latter means significant difference of the properties in different days (P < 0.05).

The color change evaluation by panelists demonstrated that the nanocomposite film protects fish fillets from discoloration. So, the uncovered fish fillets became darker than the covered ones. The protective effect on the nanocomposite coverage is attributed to the coatings' ability to reduce oxidation and microbial growth, which are primary contributors to color degradation in fish (Khorami et al., 2024). Textural evaluation of fish fillets by panelists revealed that the covered fillets retain better firmness than uncovered ones. The nanocomposite film helps to keep the fish fillets moist, which is crucial for maintaining texture. By preventing dehydration, the nanocomposite film slows down the texture damage of fillets over time (Mirza et al., 2023). The overall acceptance of fish fillets covered by the nanocomposite film during storage is affected by the protective ability of coverage for preserving sensory attributes such as color, texture, and flavor. The results of this study revealed that the overall acceptance of fish fillets was significantly affected by the nanocomposite coverage (P < 0.05).

## 4 Conclusion

This study investigated the effect of the incorporation of SNCs and NEONEs in CH/CS (62/38) film on the physical properties of chitosan/starch film. The results demonstrated that the SNCs and NEONEs incorporation into the CH/CS film decreased the water solubility, water absorption capacity, and oxygen permeability. Also, the mechanical properties of CH/CS composite film improved by the simultaneous addition of SNCs and NEONEs. The microstructural evaluation revealed the presence of strong interactions between CH/CS, SNCs, and NEONEs due to the nanocomposite film production. Coverage of the *Eleutheronema tetradactylum* fillets with the prepared nanocomposite film prevents the quality change of the fish fillets during chilled storage at 4 °C. Coverage of the fish fillets with the nanocomposite film reduced the increasing rate of peroxide value, free fatty acid production, microbial growth, T*V*B-N, and TBA index during chilled storage at 4 °C. The shelf life extension using effective protective nanocomposite film not only enhances quality but also aligns with consumer preferences, making these products more appealing in the market. The industrial application of the CH/CS/SNCs/NEONEs film represents a promising frontier in food preservation technology. This method not only enhances the shelf life of fish products but also aligns with consumer demand for natural and minimally processed foods. The results of this study are very encouraging for the employment of nanocomposite films in food packaging.

## CRediT authorship contribution statement

**Hamed Ahari:** Supervision. **Fatemeh Kalateh-Seifari:** Writing – original draft. **Shima Yousefi:** Supervision.

## Declaration of competing interest

The authors declare that they have no known competing financial interests or personal relationships that could have appeared to influence the work reported in this paper.

## Data Availability

Data will be made available on request.
